# Altered microvasculature in patients with adult-onset dermatomyositis using optical coherence tomography angiography

**DOI:** 10.1038/s41598-025-10898-y

**Published:** 2025-07-10

**Authors:** Eliane Luisa Esser, Steven Brozmann, Sebastian Dierse, Martin Dominik Leclaire, Nicole Eter, Jan Ehrchen, Natasa Mihailovic

**Affiliations:** 1 Department of Ophthalmology, University of Muenster Medical Center, Albert-Schweitzer-Campus 1, Building D15, 48149 Muenster, Germany; 2 Augen-Zentrum-Nordwest, Ahaus, Germany; 3 Department of Dermatology, University Hospital of Muenster, Muenster, Germany; 4 Department of Ophthalmology, Klinikum Bielefeld Gem. GmbH, Bielefeld, Germany

**Keywords:** Dermatomyositis (DM), Vessel densitiy, Choriocapillaris, OCTA, FAZ, Retinal thickness, Eye diseases, Skin diseases, Autoimmune diseases, Inflammatory diseases, Imaging techniques

## Abstract

To assess ocular microvascular vessel density (VD) in patients with adult-onset dermatomyositis (DM) measured by optical coherence tomography angiography (OCTA). 10 eyes of 10 patients with DM without interstitial lung disease were included in this cross-sectional study. All patients underwent ophthalmological and systemic evaluation, including nailfold capillaroscopy and OCTA examination (RTVue XR Avanti, Optovue Inc., Fremont, California, USA). The VD data of the 3 × 3 mm OCT angiogram of the superficial and deep capillary plexus (SCP, DCP), the choriocapillaris (CC) and radial peripapillary capillary density (RPC) of the optic nerve head (ONH) were extracted and analyzed. The data were compared with a gender- and age-matched healthy control group (10 eyes from 10 healthy individuals). The mean age of the patient group was 56.7 ± 3.11 years and 55.5 ± 10.76 years of the control group (*p* = 0.68) with 8 female and 2 male subjects per patient group and control group respectively. The VD in the *inside disc* area of the ONH was significantly reduced in the patient group compared to healthy controls (*p* = 0.028). A significant positive correlation was found between CC VD and nailfold capillary density (rₛₚ = 0.673, *p* = 0.033). This exploratory study identifies reduced VD in the ONH region of adult DM patients and suggests a link between ocular and systemic microvascular involvement. OCTA may offer non-invasive insights into microcirculatory changes in DM. Larger, longitudinal studies are warranted to validate these findings and evaluate the utility of OCTA-derived parameters as potential biomarkers in this rare disease.

**Supplementary Information:**

The online version contains supplementary material available at 10.1038/s41598-025-10898-y.

## Introduction

Dermatomyositis (DM), an idiopathic inflammatory disease classified within the myopathies^1^, is a rare condition with incidence estimates range from 1 to 15 per million, while prevalence estimates range from 1.2 to 21 per 100,000^2^ and a female-to-male distribution of approximately 2:1^3^. Inflammatory skin lesions like heliotrope rash and Gottron papules as well as proximal muscle involvement are the name-giving hallmarks of disease. However, the disease is not confined to skin and muscles but can affect other organs such as the lungs, the heart and the gastrointestinal tract. Moreover, there is an increased risk of malignancy especially in patients with autoantibodies like TIF-1 gamma^4^. Microvascular complement deposition is a feature of dermatomyositis pathology^5^, however, the reason for complement activation leading to microangiopathy in DM remains unclear. Due to the diversity of clinical manifestations in DM patients, vascular involvement is often under-evaluated, causing the treatment of refractory vascular damage challenging^6^. The presence of myositis specific and myositis associated antibodies strongly supports that autoimmunity is a crucial aspect of dermatomyositis pathophysiology^1,7,8^. Microvasculopathy within the capillaries of the affected muscles is an important early event in DM pathophysiology but microvascular abnormalities have also been observed in other organs^9,10^. Accordingly, in patients with DM microvascular abnormalities are observable in nailfold capillaroscopy (NFC), a common assessment tool for autoimmune connective tissue diseases. Common diagnostic findings observed in NFC in patients with DM include capillary ectasia and bushy capillaries, reduced capillary density, capillary hemorrhages, and enlarged or lost capillaries when compared to the general population, indicating prevalent microvascular disorders^8^.

Unlike NFC, optical coherence tomography angiography (OCTA) enables quantitative assessment of the ocular microcirculation, providing three-dimensional imaging of vascular structures within the retina, choroid and optic nerve head in a fast and dyeless manner. Various studies have demonstrated significantly reduced retinal vessel density (VD) in patient groups with rheumatic diseases, including juvenile DM, Sjögren’s syndrome, systemic lupus erythematodes and rheumatoid arthritis^11–14^. Furthermore, our study group recently showed that in patients with systemic sclerosis there was a positive correlation between between nailfold capillary density and VD of OCTA of the choriocapillaris, hence a correlation with disease severity^15^. These high-resolution images may therefore have the potential to contribute to the early detection of changes in microcirculation, thereby improving the diagnosis of vascular diseases^16^.

To date, only two original studies have examined ocular microvasculature in patients with DM using OCTA. Huang et al. investigated macular VD in adult DM patients with interstitial lung disease (ILD) and reported a reduction in the superficial capillary plexus (SCP). Yilmaz Tugan et al. analyzed both macular and optic nerve head (ONH) regions in *juvenile* DM and observed significant differences only in the deep capillary plexus (DCP).

However, no previous studies have investigated adult-onset DM patients without ILD, and none have evaluated ocular OCTA findings in relation to systemic microvascular alterations, such as those assessed by NFC. Given the rarity of adult-onset DM, the present study was designed as a hypothesis-generating, exploratory investigation to provide further initial insight into potential ocular vascular involvement in DM patients and potentially lay the groundwork for future longitudinal research.

## Methods

This cross-sectional study enrolled 10 eyes from 10 patients diagnosed with DM and 10 eyes from 10 healthy controls, matched for gender and age. The primary diagnosis of DM was based on clinical presentation (skin and muscle changes), histopathological findings from skin or muscle biopsies, laboratory parameters reflecting inflammatory activity, and the presence of myositis-specific antibodies^17^. None of the patients had interstitial lung disease. The control group was prospectively recruited and examined using identical imaging protocols. Control subjects consisted of healthy individuals presenting for routine ophthalmologic evaluation at our affiliated private practice, as well as healthy hospital staff volunteers.

The study received approval from the Ethics Committee of the University of Muenster and adhered to the principles outlined in the Declaration of Helsinki (2022-285-f-S). Informed consent was obtained from all participants after a thorough explanation of the examinations.

Patients displaying indications of oncological diseases as well as patients in complete disease remission were excluded. Exclusion criteria moreover encompassed individuals with media opacities affecting imaging quality, vitreoretinal diseases, prior retinal surgery, macular edema, glaucoma, or neurological disorders. Additionally, participants with any signs of retinopathy or those diagnosed with diabetes mellitus were excluded from the study. All participants underwent a comprehensive ophthalmologic examination, including refraction assessment (spherical equivalent), best-corrected visual acuity (BCVA, decimal notation), anterior segment examination, binocular fundus examination, and optical coherence tomography angiography (OCTA). Information regarding height, weight, smoking habits, and cardiovascular diseases was collected from all participants, and the control group was matched accordingly.

### Optical coherence tomography angiography

**Fig. 1:**
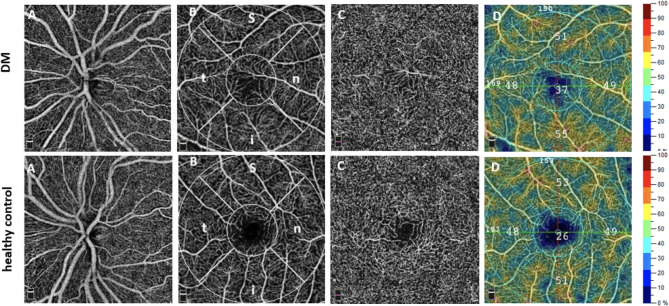
Exemplary optical coherence tomography angiography (OCTA) images of a dermatomyositis (DM) patient (top row) and healthy control (bottom row). A: OCTA angiogram of the optic nerve head (radial peripapillary capillary, *RPC*, 4.5 × 4.5 mm). B: *En face* angiogram of the macula (superficial OCTA angiogram, 3 × 3 mm displaying the analyzed subregions (outer circle = parafovea, inner circle = fovea, s = superior, i = inferior, n = nasal, t = temporal). C: *En face* images of the macula (deep OCTA angiogram, 3 × 3 mm) D: color-coded vessel density (VD) map of the superficial OCTA angiogram.

OCTA imaging for all subjects was performed using the AngioVue™ Imaging System (RTVue XR Avanti with AngioVue; Optovue Inc., Fremont, CA, USA). The split-spectrum amplitude-decorrelation angiography (SSADA) algorithm was employed to generate the OCTA data. A comprehensive description of the OCTA technology has been previously published and detailed elsewhere^18^. A 3 × 3 mm scan was used for OCTA imaging of the macula and a 4.5 × 4.5 mm scan for imaging of the optic nerve head (Fig. 1). Only OCTA images with a signal strength index (SSI) of at least 6 out of 10 were included in the study; images showing lines or gaps due to weak signal strength or motion artifacts were excluded. Multiple OCTA scans were acquired for each participant, and the highest-quality scan, as determined by SSI, image clarity, and the absence of motion or segmentation artifacts, was selected for analysis. An expert reader (EE, NM) reviewed and verified the automated segmentation before data analysis.

### Nailfold capillaroscopy

Capillaroscopy is a diagnostic technique that involves the examination of capillaries, particularly those located in the nailfold area. It is a non-invasive imaging method that allows for the visualization of the microcirculation in the capillaries near the skin surface^19,20^. Capillaroscopy is commonly used to assess microvascular abnormalities and is especially relevant in the context of autoimmune diseases and connective tissue disorders^21^.

NFC was performed in patients by the same expert examiners in patients and controls (SB and EE) in three fingers of both hands as described previously^15^. A minimum of four images were acquired for each finger. To enhance image quality, an immersion oil was applied to the nailfold using a cotton swab before capturing each image. The analysis was performed after the patients had been seated for at least 15 min, maintaining room temperature.

Using NFC the anatomy of the nailfold capillaries [capillary length, capillary lumen, hairneedle, torqueing, calibre variation, ectasia, megacapillary, bleeding, elongation, edema, branching and sludge] was evaluated semi-quantitatively. For quantitative analysis and for comparison to the OCTA data the mean capillary density per millimeter was calculated.

### Statistics

Excel 2016 was utilized by Microsoft to manage data. IBM SPSS^®^ Statistics 29 for Windows (IBM Corporation, Somers, NY, USA) was used for statistical analyses. The data was tested for normality using the Shapiro-Wilk test, and the findings indicated that they were not. Data are reported as mean ± standard deviation in order to improve comparability. The Spearman’s correlation coefficient (rSp.) was employed to represent the degree of connection between two variables, and the Mann-Whitney-U test for non-normally distributed variables was used to compare the two groups. All tests were two-tailed. A p-value < 0.05 was considered statistically significant. Given the small sample size, all analyses were considered exploratory and hypothesis-generating.

## Results

The demographic characteristics, refraction data and BCVA of both groups are presented in Table 1. No statistically significant difference in age was observed between the DM patient group and the healthy control group (*p* = 0.68). In the patient group, all 10 patients exhibited active disease, ranging from mild to moderate to severe. None of the patients had inactive disease. Disease activity was assessed based on the presence of characteristic skin lesions and/or muscle symptoms, as well as laboratory findings, including inflammatory markers and muscle enzyme levels. Detailed data are provided in Supplemetary Tables 1 and 2.

**Table 1:** Demographics and clinical characteristics of participants. Data are presented as mean values with standard deviation (SD). *N = number; m = male; f = female; dpt = diopters*.

	Patients	Controls	*p*-value
Subjects (n)	10	10	-
Eyes (n)	10	10	-
Gender (f/m)	8/2	8/2	-
Age (years)	56.7 ± 13.11	55.5 ± 10.76	0.68
Time since diagnosis (years)	2.22 ± 2.26	-	-
Spherical equivalent (dpt)	0.18 ± 1.04	−0.31 ± 1.83	0.739
Visual acuity (decimal notation)	0.88 ± 0.15	0.92 ± 1.32	0.353

### OCTA

Statistical analysis revealed no significant differences in VD between patients and controls in the SCP, DCP or the choriocapillaris (CC). The VD of the *inside disc* area of the ONH demonstrated a significant reduction in the patient group (patients: 44.47 ± 3.59; healthy controls: 50.46 ± 5.72; *p* = 0.028). Detailed VD data of the SCP, DCP, ONH, CC, and foveal avascular zone (FAZ) area are summarized in Table 2.

**Table 2:** Vessel density (VD) data of the superficial (SCP) and deep capillary plexus (DCP) of the macula, the optic nerve head (ONH), choriocapillaris (CC) and foveal avascular zone (FAZ) area. Data are presented as mean values with standard deviation. *DM = dermatomyositis; ssi = signal strength index; bold = statistically significant p-values*.

OCTA Parameter	DM Group (*n* = 10)	Control Group (*n* = 10)	*p*-value
**SCP (VD, %)**			
Whole en face	44.29 ± 2.93	43.50 ± 4.07	0.853
Fovea	18.36 ± 8.83	18.51 ± 5.95	1.000
Parafovea	46.83 ± 2.47	45.77 ± 4.55	0.481
Temporal	45.39 ± 2.64	44.94 ± 3.21	0.853
Superior	48.88 ± 2.78	46.97 ± 3.78	0.481
Nasal	44.91 ± 2.52	43.69 ± 6.44	0.796
Inferior	48.15 ± 4.06	47.51 ± 5.76	1.000
**DCP (VD**,** %)**			
Whole en face	48.08 ± 4.02	50.69 ± 3.25	0.165
Fovea	34.22 ± 9.39	36.37 ± 7.18	0.529
Parafovea	50.12 ± 3.49	52.40 ± 3.11	0.165
Temporal	51.73 ± 3.73	52.79 ± 2.58	0.796
Superior	49.97 ± 3.47	52.21 ± 3.41	0.089
Nasal	49.17 ± 4.08	52.30 ± 3.15	0.089
Inferior	49.73 ± 3.37	52.24 ± 4.58	0.190
**ONH (VD**,** %)**			
Whole en face	48.75 ± 2.72	48.41 ± 2.55	0.853
Inside disc	44.47 ± 3.59	50.46 ± 5.72	**0.028**
Peripapillary	52.90 ± 2.51	51.48 ± 2.14	0.190
**CC (VD**,** %)**			
Whole en face	69.00 ± 5.28	70.43 ± 2.03	0.971
**FAZ (mm**^2^)	0.23 ± 0.12	0.23 ± 0.10	1.000
**SSI**	7.9 ± 0.88	7.6 ± 0.70	0.579

### Nailfold capillaroscopy data and correlation with OCTA analysis

**Fig. 2:**
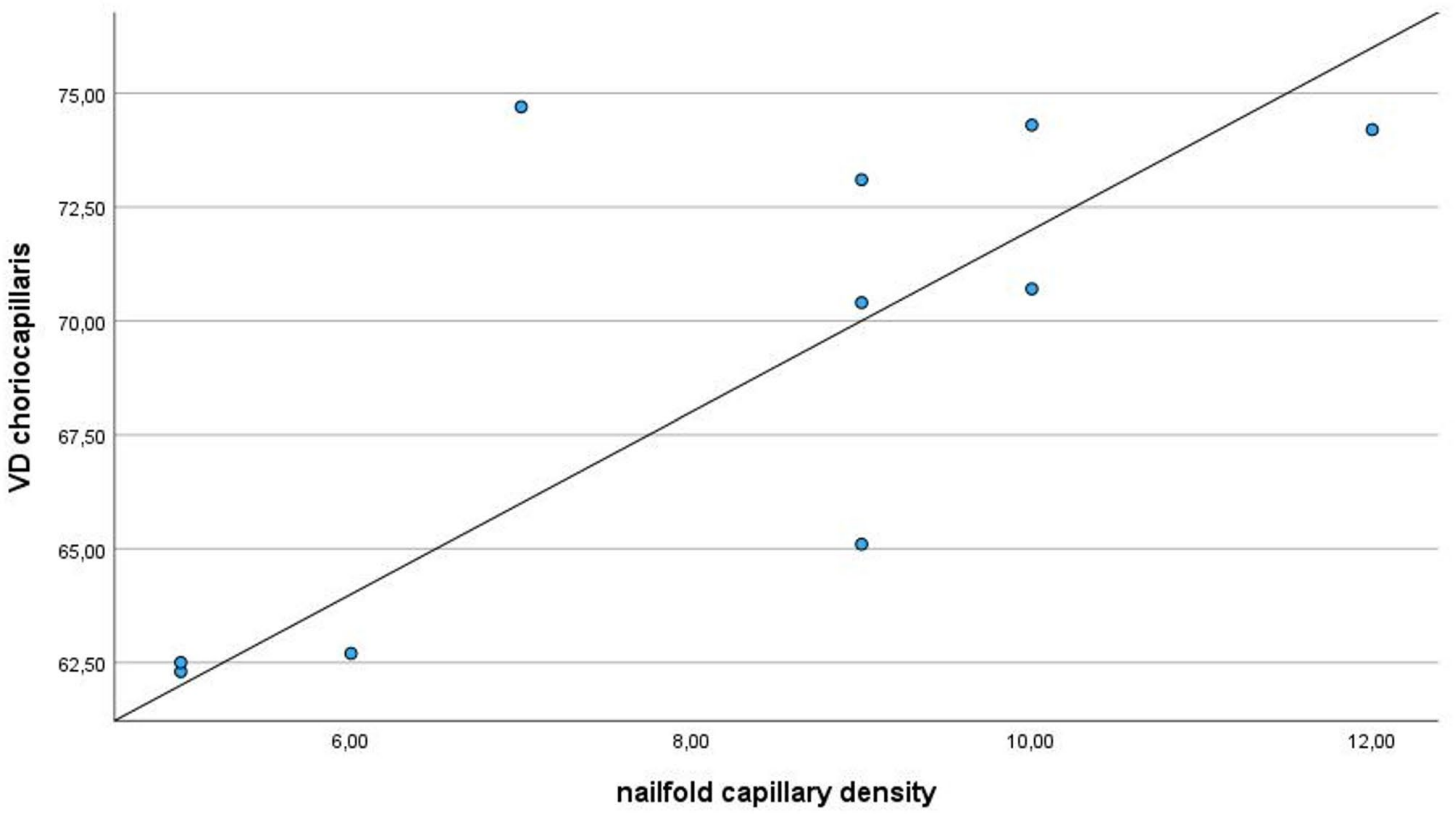
Correlation analysis revealed a significant positive relationship between choriocapillaris vessel density (*whole en face)* and nailfold capillary density (rSp = 0.673; *p* = 0.033).

A statistically significant correlation was observed between the nailfold capillary density and the VD of the CC (*p* = 0.033) (Fig. 2). The detailed NFC data and correlation data are shown in Supplementary Tables 2 and 3.

## Discussion

In this study, we observed a significantly reduced VD in the radial peripapillary capillary *(inside disc)* of the ONH in eyes of patients with active DM compared to healthy controls. To our knowledge, only two original studies and one case report have investigated the ocular microvasculature in patients with DM using the technology of OCTA^11,22,23^. The key advantages of this modern imaging modality include its non-invasiveness, ease of use, and excellent reproducibility and repeatability. In recent years, researchers have increasingly utilized OCTA-derived quantitative retinal perfusion measurements to investigate both ocular and systemic vascular conditions. Notably, OCTA has also been employed to assess retinal perfusion in autoimmune diseases, including rheumatoid arthritis, systemic lupus erythematosus, and systemic sclerosis^14,15,24^.

In their OCTA study, Huang et al. analyzed 16 adult patients with active DM and ILD, along with 16 healthy controls. Their investigation focused on the VD of the SCP and retinal thickness, while data on the DCP and ONH were not provided. Huang et al. reported a significantly reduced VD in various regions of the SCP in DM patients compared to healthy controls and concluded that OCTA is a valuable tool for evaluating ocular microvascular alterations and monitoring disease progression in DM^22^. Our findings extend the previous observations of Huang et al. by demonstrating, for the first time, a reduction in VD in the ONH region in adult patients with active DM who do not have ILD. In contrast to Huang et al., however, we did not observe significant differences in the macular region between DM patients and healthy controls. Another OCTA study investigating VD in patients with DM was conducted by Yilmaz Tugan et al., who analyzed 10 patients with juvenile dermatomyositis (JDM) and 15 healthy controls. Their data showed a reduced VD in various regions of the DCP, particularly in the parafovea and its subregions, in JDM patients compared to healthy controls. No significant differences were found in the SCP, ONH or FAZ parameters^11^. The inconsistent findings between our study and those of Huang et al. and Yilmaz Tugan et al. may be explained by several factors, including small sample sizes, differences in disease subtype (adult vs. juvenile DM), and varying degrees of systemic involvement such as the presence of interstitial lung disease in the cohort studied by Huang et al.

Previous studies have demonstrated that NFC can be a useful method for identifying microvascular changes associated with DM^25–28^. As a simple and non-invasive technique, it allows for the assessment of small vessels in the nailfold region and plays a crucial role in the diagnosis of DM, particularly by evaluating microvascular alterations in the nails and nail bed^29^. To our knowledge, none of the existing studies investigating ocular microvasculature in DM patients have included NFC data in their analyses. As expected, our nailfold capillaroscopy findings revealed characteristic alterations in DM patients, including ectasia, megacapillaries, and microhemorrhages (Supplementary Table 1). However, capillary density was not reduced in our cohort, with a mean of 9 capillaries/mm, which falls within the normal range (7–12 capillaries/mm, mean 9.1 capillaries/mm)^30,31^. While previous studies have reported slightly reduced capillary density in DM patient cohorts^32^, this reduction appears to be most pronounced within the first six months following diagnosis. In contrast, our cohort had a mean disease duration of 2.2 years, which may explain the absence of significant capillary loss. Notably, our results revealed a significant positive correlation between the nailfold capillary density and choriocapillary VD in patients with DM as assessed by OCTA. However, we did not collect NFC data in the control group, based on the assumption that capillary morphology and density are typically within physiological ranges in healthy individuals which is supported by existing literature^33,34^. We acknowledge this as a limitation of our study. To our knowledge, no published data are currently available examining correlations between nailfold capillary density and OCTA-derived VD in healthy subjects. Future research including NFC in control populations will be essential to determine whether the observed correlation is disease-specific or reflects a more general physiological association.

However, the overall literature on OCTA imaging in DM remains highly limited. Aside from the mentioned studies by Yilmaz Tugan et al. which focused on patients with JDM and Huang et al. who included adult DM patients with ILD, only one additional case report has been published. This report describes a Purtscher-like retinopathy associated with DM and utilized multimodal imaging, including OCTA, for diagnostic assessment^23^.

There are several limitations to this study that warrant consideration. First, the study employed a cross-sectional design with a small sample size, which limits generalizability. However, given the rarity of adult-onset DM, this is, to our knowledge, only the second study to investigate ocular microvasculature in *adult* DM patients using OCTA, and the first to also evaluate ONH perfusion while also correlating these findings with NFC data in these patients. Furthermore, while we took care to ensure good image quality by including only scans with an SSI ≥ 6, SSI remains a known influencing factor in OCTA analysis and may affect VD measurements^35–37^. Although refractive error was controlled for (limited from − 2.0 to + 2.0 diopters), axial length measurements were not obtained, which we acknowledge as a limitation given their potential impact on OCTA-derived VD^38^.

In summary, our study observed a significant reduction in VD within the *inside disc* region of the ONH in patients with DM compared to healthy controls, along with a positive correlation between CC VD and nailfold capillary density. These findings suggest a potential ocular microvascular involvement in patients with DM and may serve as a basis for further investigations. Nonetheless, future research with larger cohorts and longitudinal design will be important to confirm and expand upon these exploratory findings and further evaluate the potential diagnostic or prognostic relevance of OCTA in patients with DM.

## Electronic supplementary material

Below is the link to the electronic supplementary material.

Supplementary Material 1

## Data Availability

Data Availability Statement: Data are available upon request from the corresponding author.
