# Supplementary material for: Altered microvasculature in patients with adult-onset dermatomyositis using optical coherence tomography angiography
Source: Sci Rep. 2025 Jul 10;15:24995. doi: 10.1038/s41598-025-10898-y (PMC12246496; doi:10.1038/s41598-025-10898-y)
Supplement: Supplementary file 1 — Supplementary Material 1 [file 41598_2025_10898_MOESM1_ESM.docx]

| **Patient** | **Qualitative Data** | | | | | | | | | | | | | **Quantitative Data** | |
| --- | --- | --- | --- | --- | --- | --- | --- | --- | --- | --- | --- | --- | --- | --- | --- |
| **Capillary Parameters** | **Torquing** | **Caliber fluctuation** | **Ectasia** | **Mega-capillaries** | **Branches** | **Tufted capillaries** | **Elongation** | **Sludge** | **Thrombosis** | **Bleedings** | **Edema** | **Pigmentation** | **Capillary density (1000x-magnification)** | |  |
| 1 | Yes | Yes | Yes | No | No | No | No | Yes | No | Yes | No | No | 9 | |  |
| 2 | No | Yes | Yes | No | No | No | Yes | Yes | No | Yes | No | No | 5 | |  |
| 3 | No | No | Yes | No | No | No | No | No | No | No | No | No | 10 | |  |
| 4 | Yes | Yes | No | No | No | Yes | Yes | Yes | No | No | No | No | 9 | |  |
| 5 | Yes | Yes | Yes | Yes | Yes | Yes | Yes | Yes | No | Yes | No | No | 5 | |  |
| 6 | No | Yes | Yes | No | Yes | No | No | No | No | No | No | No | 10 | |  |
| 7 | Yes | Yes | Yes | No | Yes | No | No | Yes | No | Yes | No | No | 6 | |  |
| 8 | No | No | Yes | No | No | No | No | No | No | No | No | No | 7 | |  |
| 9 | Yes | Yes | No | No | Yes | Yes | Yes | Yes | No | No | No | No | 12 | |  |
| 10 | No | No | No | No | No | Yes | No | No | No | No | No | No | 9 | |  |

**Supplementary Table 1.** Nailfold capillary microscopy overview of the qualitative and quantitative data of the patients.

| **Patient** | **Myosits-specific Antibodies** | | | | | | | **Myositis-related Antibodies** | | | **Rheumatological Control Date** | | | |
| --- | --- | --- | --- | --- | --- | --- | --- | --- | --- | --- | --- | --- | --- | --- |
|  | **Mi-2** | **TIF-1-Gamma** | **NXP2** | **ASA** | **MDA5** | **Anti-SAE** | **Anti-Jo-1** | Anti-PM-Scl-7 | Anti-CENB | Anti-PL-12 | CRP (mg/dl) | CK (U/I) | LDH (U/I) | ANA |
| 1 | No | **Yes** | No | No | No | **Yes** | No | **Yes** | No | No | <0.5 | 63 | 214 | 1:160 |
| 2 | No | **Yes** | No | No | No | No | **Yes** | No | **Yes** | No | <0.5 | 46 | 469 | 1:2560 |
| 3 | No | No | No | No | No | No | No | No | No | No | <0.5 | 100 | 250 | 1:1520 |
| 4 | No | **Yes** | No | No | No | No | No | No | No | No | <0.5 | 93 | 207 | 1:160 |
| 5 | No | **Yes** | No | No | No | No | No | No | No | **Yes** | <0.5 | 91 | 241 | 1:160 |
| 6 | No | No | **Yes** | No | No | No | No | No | No | No | <0.5 | 384 | 715 | 1:1520 |
| 7 | No | No | No | No | **Yes** | No | No | No | No | No | <0.5 | 73 | 275 | 1:1520 |
| 8 | No | No | No | No | **Yes** | No | No | No | No | No | <0.5 | 88 | 271 | 1:80 |
| 9 | No | No | **Yes** | No | No | No | No | No | No | No | <0.5 | 23 | 222 | 1:80 |
| 10 | No | **Yes** | No | No | No | No | No | No | No | No | <0.5 | 260 | 221 | 1:160 |

**Supplemetary Table 2.** Laboratory findings of the included patients.

|  | **OCTA** | **p-value** | **r-Sp** |
| --- | --- | --- | --- |
| **SCP (VD)** | Whole en Face | 0.480 | 0.253 |
|  | Foveal | 0.385 | 0.309 |
|  | Parafoveal | 0.645 | 0.167 |
|  | Temporal | 0.746 | -0.118 |
|  | Superior | 0.561 | 0.210 |
|  | Nasal | 0.959 | -0.019 |
|  | Inferior | 0.826 | 0.080 |
| **DCP (VD)** | Whole en Face | 0.101 | 0.548 |
|  | Fovea | 0.159 | 0.482 |
|  | Parafovea | 0.109 | 0.537 |
|  | Temporal | 0.140 | 0.502 |
|  | Superior | 0.153 | 0.488 |
|  | Nasal | 0.064 | 0.605 |
|  | Inferior | 0.267 | 0.389 |
| **CC** | Whole en face | **0.033** | 0.673 |
| **ONH (VD)** | Whole en Face | 0.301 | -0.364 |
|  | Inside Disc | 0.420 | -0.288 |
|  | Peripapillary | 0.267 | -0.389 |
| **FAZ** |  | 0.087 | -0.568 |

**Supplementary Table 3.** Correlation between the nailfold capillary density as assessed in the nailfold and the vessel density (VD) of the superficial (SCP) and deep (DCP) OCT angiogram of the macula, the optic nerve head (ONH) and the foveal avascular zone (FAZ) area in the patient group; *Nailfold capillaroscopy (NFC); rSp = Spearman correlation coefficient; bold = statistically significant p-values*
